# Collateral Victim or Rescue Worker?—The Role of Histone Methyltransferases in DNA Damage Repair and Their Targeting for Therapeutic Opportunities in Cancer

**DOI:** 10.3389/fcell.2021.735107

**Published:** 2021-11-16

**Authors:** Lishu He, Gwen Lomberk

**Affiliations:** ^1^Department of Pharmacology and Toxicology, Medical College of Wisconsin, Milwaukee, WI, United States; ^2^Division of Research, Department of Surgery, Medical College of Wisconsin, Milwaukee, WI, United States; ^3^Genomic Sciences and Precision Medicine Center, Medical College of Wisconsin, Milwaukee, WI, United States; ^4^LaBahn Pancreatic Cancer Program, Medical College of Wisconsin, Milwaukee, WI, United States

**Keywords:** cancer epigenetics, chromatin, epigenetic drugs, histone methyltransferases, DNA damage repair (DDR)

## Abstract

Disrupted DNA damage signaling greatly threatens cell integrity and plays significant roles in cancer. With recent advances in understanding the human genome and gene regulation in the context of DNA damage, chromatin biology, specifically biology of histone post-translational modifications (PTMs), has emerged as a popular field of study with great promise for cancer therapeutics. Here, we discuss how key histone methylation pathways contribute to DNA damage repair and impact tumorigenesis within this context, as well as the potential for their targeting as part of therapeutic strategies in cancer.

## Introduction—The Accident Scene

Since the discovery of DNA by Swiss scientist Friedrich Miescher in 1869, numerous researchers have expanded on his work and contributed to our understanding of how genetic information was encoded, preserved, and stably transmitted across generations by DNA (Ciccia and Elledge, 2010). Now we know that DNA is liable to change, as it is subject to extensive lesion formation due to constant genomic insults from endogenous and exogenous sources such as hydrolysis, oxidation, ionizing radiation, UV radiation, and various chemical agents among others (Friedberg et al., 2006; Hakem, 2008; Chatterjee and Walker, 2017). If not repaired timely and correctly, these lesions may damage genome maintenance machinery, trigger mutagenesis, and threaten cell integrity.

The explosion of the field of DNA damage response (DDR), however, came long after Watson and Crick published their theory of the DNA double helix in the 1950s (Watson and Crick, 1953; Friedberg et al., 2006). Various mechanisms through which DNA damage is resolved on a molecular level have been identified. The most notable discoveries are those by the three pioneers in DNA repair who shared the 2015 Nobel Prize in Chemistry: Tomas Lindahl for establishing the role of DNA glycosylase enzymes in base excision repair (BER) (Krokan and Bjørås, 2013), Paul Modrich for mismatch repair (MMR) (Lahue et al., 1989), and Aziz Sancar for defining the reversal of ultraviolet damage to DNA by nucleotide excision repair (NER) (Petit and Sancar, 1999).

When DNA lesions remain unrepaired, they may accumulate and block DNA replication progression, resulting in double-stranded breaks (DSBs) that are more difficult to repair and far more toxic (Khanna and Jackson, 2001; Jackson and Bartek, 2009). So far, studies have elucidated two umbrella pathways of DSB repair, namely, homologous recombination (HR) and non-homologous end-joining (NHEJ). HR utilizes template DNA and exchanges of equivalent DNA regions between homologous chromosomes to promote high-fidelity DSB repair, preserving genomic stability (Sung and Klein, 2006; Tubbs and Nussenzweig, 2017). NHEJ, on the other hand, is more prone to deletions and insertions as it ligates broken DNA ends without a template (Lieber, 2008; Tubbs and Nussenzweig, 2017). The initial detection of DNA damage and cellular response that triggers cell cycle arrest to allow repair or undergo cell death is coordinated by various DNA damage signaling cascades, contributing to the cells’ ability to defend against potential mutagenesis (Hanawalt, 2015). These damage sensing pathways are extensively reviewed in Jackson and Bartek (2009), Ciccia and Elledge (2010), Hanawalt (2015). Together, the DNA damage sensing and DDR pathways are the core molecular machinery responsible for rescuing the cell from deleterious effects of DNA damage and evading pathobiology outcomes, such as cancer.

With recent advances in understanding the human genome and gene regulation in the context of DNA damage, epigenetics has emerged as a rapidly growing field with great promise for therapeutics. Epigenetics refers to the study of reversible, heritable changes in genome function that occur without alterations to the DNA sequence (Russo et al., 1996). Different epigenetic changes, such as DNA methylation and histone post-translational modifications (PTMs) or “marks,” regulate gene expression and underlie both healthy and diseased human physiology. Nucleosomes, organized modules of duplexed DNA wrapping around histones, serve as the basic units for chromatin, which is the fundamental packaging structure of all eukaryotic genomes (Li et al., 2007). It can be “closed” or “open,” generally associating with transcription repression and activation, respectively (Gillette and Hill, 2015). There is dynamic regulation of chromatin to modify accessibility to DNA during processes such as replication, transcription, DDR, and more (Hanawalt, 2015). Chromatin marks impact DNA accessibility by modulating histone-DNA interactions and serving as docking sites for “reader” proteins, dedicated effectors of those PTMs to achieve their specific transcriptional outcome (Musselman et al., 2012; Gillette and Hill, 2015). In addition to the reader proteins, other proteins involved in altering chromatin status include “writer” proteins, which are enzymes that add PTMs to histones, and “erasers,” which remove these marks (Musselman et al., 2012). Furthermore, epigenetic modifications can facilitate responses to DNA damage. Conceptually, during DNA damage, epigenetic marks can occur in a collateral event either as part of the chromatin disruption in the wake of DNA damage or as a flag to identify a genomic region that requires repair. Alternatively, the role of epigenetic marks during DDR processes can be a more active function that mechanistically contributes to rescue the cell from DNA damage. Because of the dynamic functions of histone PTMs and their mediators, they have been increasingly considered as therapeutic targets by the pharmaceutical industry and academic pharmacologists. Here, we discuss how chromatin modifiers and their histone modifications, specifically key histone methylation pathways, contribute to DNA damage repair and impact tumorigenesis within this context, as well as the potential for their targeting as therapeutic strategies in cancer.

## Lights and Sirens—The Chromatin Response in DNA Damage Repair

While various players in the DDR pathways have long been studied, there is a rapidly growing understanding that higher-order chromatin also significantly impacts DDR via remodeling and post-translational modification from the moment DNA damage strikes. In other words, the chromatin landscape is not only pivotal to the regulation of epigenetic changes for healthy human physiology, but also significant to our heritable cell machinery for DNA transcription, replication, and repair. To protect against endogenous and exogenous agents from causing deleterious DNA damage, chromatin controls accessibility to the genetic material it packages as well as facilitates swift recognition and precise repair of any damage that has occurred (Kouzarides, 2007).

Among the known histone PTMs, acetylation, phosphorylation, and ubiquitylation are among some of the most widely studied participants in key DDR pathways (Gong and Miller, 2013). For example, the histone variant H2AX is phosphorylated on Ser139 upon DNA damage by the phosphatidylinositol-3 kinase (PIKK) family proteins that include key DDR pathway components, such as ataxia telangiectasia mutated (ATM), DNA-dependent protein kinase (DNA-PK), as well as ATM and RAD3-related (ATR) (Fernandez-Capetillo et al., 2002; Bonner et al., 2008; Gong and Miller, 2019). The phosphorylated H2AX is then recognized by MDC1, triggering a downstream ubiquitylation cascade via the recruitment of RNF8, a RING-domain ubiquitin ligase (Stucki et al., 2005; Ciccia and Elledge, 2010; Bekker-Jensen and Mailand, 2011). RNF168, another ubiquitin ligase, binds and amplifies the RNF8-initiated cascade and leads to recruitment of chromatin-associated genome caretakers that assist in DSB repair (Bekker-Jensen and Mailand, 2011). MDC1 also recruits NuA4 and the histone acetyltransferase TIP60, whose activity contributes to local chromatin relaxation, ATM activity, and DNA damage-induced phosphorylation (Bonner et al., 2008; Xu and Price, 2011; Gong and Miller, 2019). Here, we will focus on histone methylation as well as their modifiers in the various DDR pathways and provide an overview of applications of histone methyltransferase inhibitors within the context of DDR as novel therapeutic strategies for cancer.

First discovered in the 1960s (Murray, 1964), histone methylation has been identified as a critical modulator of DDR pathways, and studies exploring this role have been steadily increasing. Mostly known for their function in transcriptional regulation, histone methylation occurs as mono- (me), di- (me2) or tri- (me3) methyl groups on the ε-amino group of lysine residues, as well as in a mono- (me), di-symmetrical (me2s), or di-asymmetrical (me2a) state on arginine residues (Kouzarides, 2002; Greer and Shi, 2012; Gong and Miller, 2019). These reactions are catalyzed by histone methyltransferases (HMTs) through transferring methyl groups donated by S-adenosyl methionine (SAM) to their target residues (Murray, 1964; Greer and Shi, 2012; Gong and Miller, 2019). So far, three major groups of HMTs have been identified: SET domain-containing lysine methyltransferases (KMTs), Dot1-Like KMT with no SET domain, and protein arginine methyltransferases (PRMTs) (Okada et al., 2005; Black et al., 2012; Yang and Bedford, 2013). Apart from core histone proteins, HMTs can also methylate free histones and non-histone proteins, such as p53, TAF10, VEGFR1, among others (Murray, 1964; Kouzarides, 2002; Chuikov et al., 2004; Okada et al., 2005; Bekker-Jensen and Mailand, 2011; Xu and Price, 2011; Black et al., 2012; Greer and Shi, 2012; Yang and Bedford, 2013). The diverse array of various methylation events possesses tremendous regulatory power and frequently contributes to human diseases, including cancers (Bonner et al., 2008; Greer and Shi, 2012; Yang and Bedford, 2013).

**FIGURE 1:**
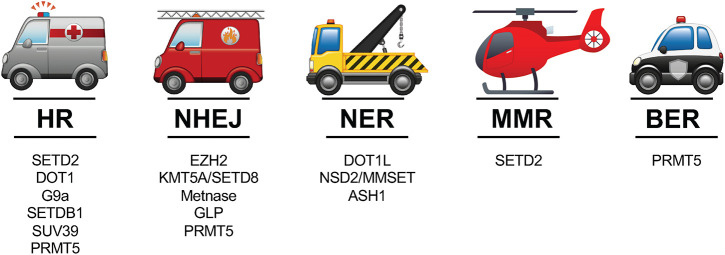
The 5 major DDR pathways and prominent HMTs involved in the context of those distinct pathways. See text for references of studies highlighting the various roles of HMTs in these pathways.

One of the first and most notable discoveries illuminating a relationship between histone methylation and DNA damage was that methylation of histone H3 Lys 79 (H3K79) promotes chromatin accumulation of p53-binding protein 1 (53BP1), a key mediator of DDR, through its Tudor domain (Huyen et al., 2004). Many HMTs, including but not limited to those for H3K4, H3K9, H3K27, H3K36, H3K79, and histone H4 lysine 20 (H4K20), have since been found to rapidly work to mark histones at sites of DNA damage and modulate DDR machinery. Following, we review our current understanding of some prominent HMTs within the context of distinct DDR pathways (Figure 1).

## Emergency Dispatch of First Responders—The Relationship of Chromatin and Epigenetic Regulators in DNA Damage Repair

### Homologous Recombination—Tending to Damage With a Stretch(er) of DNA

HR, which is the more faithful mechanism of the two for repair of DSBs, utilizes homologous DNA sequences as templates to repair damaged DNA (Sung and Klein, 2006; Tubbs and Nussenzweig, 2017). HR occurs mainly after DNA replication in the S and G2 phases of the cell cycle when homologous sister chromatids are readily available to serve as repair templates (Sung and Klein, 2006; Hustedt and Durocher, 2017). It is initiated when 3′ single-stranded DNA (ssDNA) overhangs are generated from DSB resection by helicases and nucleases. The RAD51 recombinase then assembles onto the ssDNA overhangs and recruits its template for repair DNA synthesis by invading homologous duplex DNA (Chapman et al., 2012). During the process of HR, the steps from resection to duplex DNA invasion through DNA synthesis and final resolution require transient yet extensive disruption and restoration of chromatin structure in a rapid manner.

The activities of many histone methylation marks and their respective HMTs change during the HR process. For example, tri-methylated H3 lysine 36 (H3K36me3), mediated by SETD2/HYPB methyltransferase, was established by Carvalho et al. (2014) and Pfister et al. (2014) to be required for HR repair, to promote the formation of presynaptic RAD51 filaments and subsequent loading of RAD51 to resected DNA ends. The H3K79 histone methyltransferase DOT1L (Disruptor of telomeric silencing 1-like) and some H3K9-specific KMTs, such as SETDB1, and its reader protein, HP1 (Heterochromatin Protein 1), among others, were also discovered to play pivotal roles to facilitate the recruitment of 53BP1 to DSBs and direct HR (Alagoz et al., 2015). DOT1L is structurally unique because it does not contain a SET domain that is evolutionarily conserved among KMTs (Okada et al., 2005). So far, DOT1L has been demonstrated to regulate several molecular processes, including but not limited to telomeric silencing, gene transcription, and most notably, DNA damage repair (Kari et al., 2019). Huyen et al. (2004) and Ljungman et al. (2017) suggested that DOT1L-mediated H3K79me2 marks, otherwise hidden in chromatin under normal conditions, can modify binding by the tandem tudor domain of the human 53BP1 protein after nearby DSB induction. Similar findings were found in colorectal cancer, where DOT1L-mediated H3K79me is required for chromosome structure maintenance, DNA damage checkpoint, and cell recovery via HR (Ljungman et al., 2017; Wood et al., 2018).

There are several methyltransferases that interact with and modify H3K9. The euchromatic histone-lysine *N*-methyltransferase 1/2 complex, also known as GLP/G9a, catalyzes H3K9 mono- and di-methylation and is associated with transcriptional repression (Tachibana et al., 2008). These two components can be phosphorylated by ATM, and their disruption leads to genomic instability, suggesting a role in DDR (Agarwal and Jackson, 2016; Ginjala et al., 2017). G9a is recruited to chromatin and interacts with replication protein A (RPA), a heterotrimeric protein complex that directly participates in HR by binding to the 3′ ssDNA tails and stimulating end resection (Yang et al., 2017). This interaction also modifies Rad51 foci formation, allowing for efficient HR (Gasior et al., 1998; Raderschall et al., 1999). GLP localization at DNA break sites is largely dependent on G9a (Tachibana et al., 2005, 2008). Interestingly, GLP activity on its own, for example GLP-catalyzed H4K16 methylation, was found to contribute to NHEJ (see below). SETDB1 and SUV39, two methyltransferases that tri-methylate H3K9, work with BRCA1 and HP1 to promote HR integrity by ensuring repositioning of 53BP1 to extend resection during HR in cells at G2 phase (Alagoz et al., 2015).

Compared to KMTs, PRMTs and their associated histone arginine methylation marks are not as well understood in these processes. So far, 9 PRMTs have been characterized, producing 3 categories of methylarginines: monomethylarginine, asymmetric dimethylarginine (ADMA), and symmetric dimethylarginine (SDMA) (Bedford, 2007). Type I PRMTs (PRMT1-4, 6, and 8) catalyze formation of MMA and ADMA, while type II PRMTs (PRMT5, 7, and 9) catalyze production of MMA and SDMA (Bedford, 2007; Gong and Miller, 2019). Protein arginine methylation is abundant in modifying signal transduction, gene transcription, DNA repair, mRNA splicing, and more (Yang and Bedford, 2013). PRMTs have also been linked to carcinogenesis and metastasis of various cancers. PRMT5 is a type II methyltransferase that mediates MMA or SDMA of residues such as H3R8, H2AR3, H3R2, and H4R3 (Bedford, 2007). Recent studies have reported that depletion or inhibition of PRMT5 impairs HR, inducing DNA damage accumulation, cell cycle arrest, and eventually cell death. Mechanistically, PRMT5 depletion or inhibition potentially mediates this by inducing abnormal splicing of TIP/KAT5, a DNA repair factor, or disrupting methylation of KLF4, a transcriptional regulator modulating DNA end resection, HR efficiency and TIP60 expression (Hamard et al., 2018; Checa-Rodríguez et al., 2020). Overall, there are many studies that suggest an active role of HMTs in rescuing DSB via HR (Figure 1), although the mechanisms underlying this involvement require further elucidation.

### Non-homologous End-Joining —Putting Out Destructive Fires Rapidly

NHEJ, the other main repair pathway for eukaryotic DSBs, was first named by Moore and Haber in 1996 (Moore and Haber, 1996; Lieber, 2008; Yamagishi and Uchimaru, 2017). NHEJ is referred to as “non-homologous” because in this pathway, contrary to HR, the broken ends are directly ligated without any homologous template (Lieber, 2008). Complementary to HR, NHEJ is generally favored for DDR occurring in the G1 phase of the cell cycle (Lieber, 2008; Chapman et al., 2012; Hustedt and Durocher, 2017). In this pathway, the Ku70/80 heterodimer rapidly binds to the breakage site and forms a complex with the DNA, thereby acting as a node for docking of nuclease, polymerase and ligase components (Lieber, 2008). Subsequently, the catalytic subunit of DNA-PK (DNA-PKcs), responsible for maintaining the broken DNA ends in close proximity, is recruited and activated in preparation to engage other end-processing factors and eventually re-join the broken ends (Lieber, 2008; Hustedt and Durocher, 2017). Finally, re-ligation of the broken DNA ends occurs via the DNA ligase complex. Among the many HMTs involved in NHEJ, we summarize knowledge in this context for EZH2, KMT5A, Metnase, GLP, and PRMT5 (Figure 1).

EZH2 (Enhancer of Zeste 2) is an H3K27 methyltransferase and essential component of polycomb repressive complex 2 (PRC2), regulating cell sensitivity to DNA damage via alteration of chromatin architecture as well as expression of many functional genes that participate in lineage specification, cell cycle regulation, and DNA repair (Yamagishi and Uchimaru, 2017). EZH2 has been demonstrated to interact directly with the NHEJ-related protein, Ku80. Ku80 bridges EZH2 to DNA-PK complexes, facilitating phosphorylation of EZH2 by DNA-PK and subsequent modulation of EZH2 methyltransferase activity as well as its target gene expression (Wang et al., 2016).

KMT5A, also known as SETD8, is a SET domain containing methyltransferase of H4K20 mono-methylation, which is required before di-methylation and tri-methylation can take place [54]. Although it is primarily known to use H4K20 as a substrate, evidence also exists to indicate that KMT5A interacts with non-histone proteins (Checa-Rodríguez et al., 2020). It has been shown to be vital during replication, transcription, and chromosome segregation (Moore and Haber, 1996). Like DOT1L, KMT5A function also affects 53BP1 recruitment to DSBs. Although depletion of SETD8 only moderately decreased HR efficiency in human osteosarcoma cells, the same experiment resulted in a severe abrogation in NHEJ (Dulev et al., 2014), suggesting that SETD8 promotes DSB repair via the NHEJ pathway.

Metnase is a DNA repair protein with a unique fusion of a SET domain, a nuclease domain, and a transposase/integrase domain (Lee et al., 2005). Fnu et al. (2011) found that Metnase enhances NHEJ following DSBs and that its SET domain is required for Metnase activities in DNA damage. Metnase at DSBs can also directly dimethylate H3K36, which then recruits and stabilizes DNA repair proteins at the breakage site, facilitating NHEJ efficiency (Lee et al., 2005; Fnu et al., 2011).

Although GLP localization to DNA break sites largely depends on G9a (Tachibana et al., 2005, 2008), which together are responsible for the H3K9me2 mark, GLP-catalyzed H4K16me1 is also part of its response to DNA damage (Lu et al., 2019). GLP-catalyzed H4K16me1 levels drastically increase during the early stages of DDR and cooperate with H4K20me2 to facilitate 53BP1 recruitment, favoring NHEJ-mediated DDR. The fact that GLP is commonly studied in conjunction with G9a often complicates experimental interpretations. For example, previous studies have shown that pharmacological inhibition of G9a/GLP hindered HR with contentious impact on NHEJ (Fukuda et al., 2015; Ginjala et al., 2017). However, GLP knockdown alone impaired 53BP1 foci formation and NHEJ following damage induction, while G9a knockdown only had a mild effect on NHEJ but caused severe HR defects in reporter assays (Fnu et al., 2011). This suggests G9a and G9a-catalyzed H3K9 methylation may be more significant in facilitating HR, while GLP and GLP-catalyzed H4K16me1 play a greater role in NHEJ.

In terms of PRMT involvement in this DDR pathway, Hwang et al. found that PRMT5 regulates NHEJ via methylation and stabilization of 53BP1 to promote cell survival (Hwang et al., 2020). Interestingly, these investigators also discovered a regulatory mechanism in which Src kinase phosphorylates PRMT5 at residue Y324 to suppress PRMT5 activity and block NHEJ repair. Thus, along with the aforementioned studies implicating participation of PRMT5 in HR, it is likely that PRMT5 is involved in both main pathways, possibly through 53BP1 modulation. Further studies are needed to characterize its overall involvement in DDR and determine whether favoring one pathway over another depends on specific molecular contexts, such as elevated Src activity.

### Nucleotide Excision Repair—Towing Away Helix-Distorting Lesions

Different DDR pathways are instigated to resolve damage in a substrate-dependent manner (Chatterjee and Walker, 2017). NER plays a significant role to remove helix-distorting DNA lesions such as bulky chemical adducts and photoproducts produced by UV light (Thoma, 1999; Gong et al., 2005). It is a “broad spectrum” DDR pathway, repairing a wide range of DNA damage. Mechanistically, NER acts via dual incisions on both sides of the target lesions by two different nucleases, one at the 3′ end followed by the other at the 5′ end (Petit and Sancar, 1999). After targets are removed, new DNA is synthesized to fill the gap using the complementary strand as a template and ligated back into the segment (Petit and Sancar, 1999; Gong et al., 2005).

The chromatin organization via histone displacement often impacts how cells detect and repair lesions as well as other DNA-dependent reactions. As NER acts primarily in the context of DNA, this chromatin organization becomes an important process during NER initiation (Fukuda et al., 2015; Hwang et al., 2020). For repair processes like NER to happen efficiently, lesion detection mechanisms need to activate both NER and chromatin remodeling. Previous studies have linked UV-induced histone acetylation prior to NER with increased accessibility to repair proteins, at least in the more condensed regions of chromatin (Teng et al., 2002; Gong et al., 2005). Evidence has also emerged to support the involvement of a few histone methylation pathways (Figure 1), such as DOT1L-mediated H3K79, NSD2-catalyzed H4K20, and ASH1L-mediated H3K4 methylation, in this process, which are discussed below.

Interestingly, Dot1, the yeast homolog of the methyltransferase for H3K79 methylation, also has roles in NER apart from its aforementioned involvement in HR. Dot1 promotes transcriptional restart of paused RNA polymerases following NER completion and ensures proper replication timing during the cell cycle (Chaudhuri et al., 2009; Ljungman et al., 2017). Yeast cells with H3K79R mutations and Dot1 mutations were found to be hyper-sensitive to UV light and have affected NER (Chaudhuri et al., 2009). The exact mechanisms by which Dot1 and H3K79 methylation impact NER, however, remain to be elucidated.

NSD2 and ASH1L are two additional histone methyltransferases that have been identified as players in genome-wide NER activity (Gong et al., 2005; Gillet and Schärer, 2006; Balbo Pogliano et al., 2017). NSD2 (nuclear receptor SET domain 2), also known as Multiple Myeloma SET (MMSET) or Wolf-Hirschhorn Syndrome Candidate 1 (WHSC1), is a SET domain containing KMT that primarily catalyzes di- and tri-methylation of H3K36, which regulates crucial developmental genes as well as modulates DSB repair (Nimura et al., 2009). Notably, NSD2 has also been found to catalyze H4K20 methylation and subsequent 53BP1 accumulation at DNA damage sites upon DSB induction (Pei et al., 2011). Previous studies by Chitale and Richly reported the collaboration between NSD2 and the endonuclease DICER, which facilitates heterochromatin formation and generates small non-coding RNAs that include the sequence of the damaged locus (Chitale and Richly, 2018). They later reported a mechanism by which NSD2 relocates to chromatin in a DICER-dependent manner and sets the stage for H4K20me2 to recruit XPA, a protein that binds to damaged DNA and acts as a scaffold for other repair proteins, at sites of UV lesions for genome-wide NER (Thoma, 1999; Pei et al., 2011; Chitale and Richly, 2018).

ASH1L (Absent, Small, or Homeotic discs 1-Like) is a member of the *trithorax* transcriptional regulators crucial for normal development, organ function, fertility, euchromatin formation, and ongoing transcription (Balbo Pogliano et al., 2017). It performs its regulatory functions by histone modification and chromatin remodeling via methylation of H3K4 and H3K36 (Balbo Pogliano et al., 2017). Balbo Pogliano et al. (2017) have reported a helper role of ASH1L for effective genome-wide NER. They found that DDB2, a specialized damage sensor, promotes excision of mutagenic lesions from UV damage by recruiting ASH1L, which methylates H3K4 and in turn facilitates docking of downstream NER effectors to ensure uninterrupted repair activity. Without ASH1L, the otherwise effective handoff between different NER damage recognition factors would be disrupted (Balbo Pogliano et al., 2017; Gsell et al., 2020). Thus, as with other types of DDR, histone methylation seems to have a clear role in the repair of helix-distorting DNA lesions, necessitating additional investigations to understand the full breadth of this involvement.

### Mismatch Repair—Evacuating Mis-Incorporated Bases

MMR helps protect genome integrity at replication by removing mis-incorporated bases and insertion-deletion mis-pairs from newly synthesized daughter DNA strands (Li et al., 2016). In humans, MMR starts with mismatch recognition on newly synthesized strands by the heterodimer complexes hMutSα or hMutSβ, which lead to the excision of the mismatch by an exonuclease, EXO1 (Hombauer et al., 2011; Li et al., 2016). As a result, a single-stranded DNA gap is generated, filled, and ligated, concluding the repair (Hombauer et al., 2011; Li, 2013). The most notable HMT-MMR interaction is that of SETD2 and hMutSα (Figure 1). SETD2 tri-methylates H3K36, which is required *in vivo* to recruit hMutSα onto chromatin for downstream repair (Li et al., 2013). Cells depleted of SETD2 display characteristics of MMR-deficient cells, suggesting a significant role of H3K36me3 in proper MMR progression (Li, 2013; Li et al., 2013, 2016). However, much remains to be discovered in terms of how chromatin states and their modifiers modulate the coupled MMR and replication processes.

### Base Excision Repair—Chasing Down Damaged Nucleotides

BER resolves endogenous DNA damage raised from deamination, oxidation and alkylation by removing damaged bases without causing excessive distortion to the DNA helix (Krokan and Bjørås, 2013). Protecting against various detrimental processes such as cancer, aging, and neurodegeneration (Jeppesen et al., 2011; Krokan and Bjørås, 2013), much of BER occurs in the nuclei, as well as mitochondria, and is a highly conserved system from bacteria to human. The process is initiated by one of at least 11 distinct DNA glycosylases, such as 8-Oxoguanine-DNA glycosylase (OGG1), that recognizes and removes a damaged base by cleaving its N-glycosyl bond to allow its subsequent release (Jeppesen et al., 2011). Following excision, an abasic, apurinic/apyrimidinic site, known as an AP site, remains in the absence of the base. AP endonuclease 1 (APE1) then recognizes the AP site and cleaves its backbone to produce an intermediate that is properly processed through BER (Jeppesen et al., 2011; Yu et al., 2012). Within BER, (short-patch) (SP-)BER only adds one nucleotide to the 3′-end of the cleaved AP site, followed by Pol β which helps produce a nick sealable by X-ray repair cross-complementing protein 1 and Ligase IIIα (XRCC1/Ligase IIIα) (Sobol et al., 1996; Demple and Sung, 2005). On the other hand, (long patch) LP-BER utilizes Pol β in a different manner, generating a short DNA flap cleaved by flap endonuclease 1 (FEN1) and sealed by DNA ligase I (Sobol et al., 1996; Jeppesen et al., 2011).

To date, there have been few studies illuminating effects of HMTs and their methylation marks in BER (Figure 1). The most notable HMT studied in the context of BER is PRMT5. Recent studies by Zhou et al. (2018) identified interactions between symmetrical dimethylarginine of histone H4 (H4R3me2s), catalyzed by PRMT5, and OGG1, the glycosylase initiating BER activities. OGG1 directly interacts with PRMT5, affecting its binding to histone H4 and thereby regulating H4R3me2s levels. FEN1 binds to symmetrically dimethylated H4R3, which was found to enhance its substrate binding, thereby increasing its efficiency in BER (Zhou et al., 2018). Depletion of PRMT5 decreased OGG1 activity, BER efficiency and cell survival ratio *in vitro*, suggesting that H4R3me2s can be an important downstream factor of PRMT5 function in DDR, and the PRMT5-H4R3me2s relationship bridges endogenous lesion detection by OGG1 and downstream repair. Similar to MMR, the identification of a role for PRMT5 in BER only indicates the beginning of this intriguing new area revealing the involvement of HMTs in a wide range of DDR pathways.

## Utilizing a Safety Harness—Targeting DNA Damage Response via Histone Methyltransferases in Cancer for Improved Treatment Strategies

**FIGURE 2:**
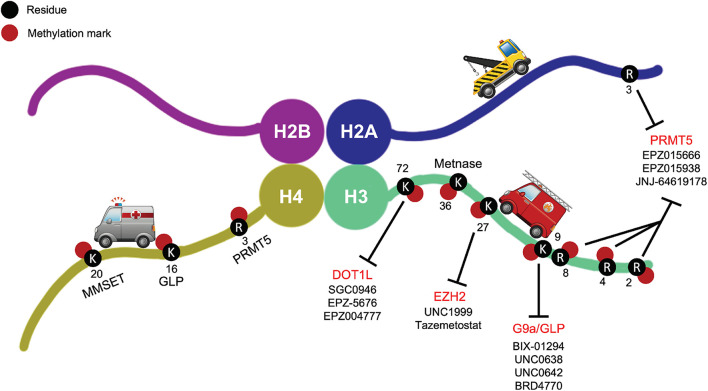
Small molecule inhibitors of DOT1L, G9a/GLP, EZH2, and PRMT5 shown with their respective target methylation marks on the histone tails. These are examples of prominent small molecule HMT inhibitors in pre-clinical testing or early stages of clinical trials as part of promising standalone or combinatorial cancer treatments.

DNA damage, as well as inadequate DDR, is one of the main causes for genomic instability and tumorigenesis. However, these very defects in DDR mechanisms serendipitously also offer therapeutic opportunities to cause lethality in cancer cells while sparing normal ones. Cancer researchers have recently popularized the term “synthetic lethality,” originally described back in 1922 and later coined in 1946, for phenomena where disruption of one gene maintains cell viability, but the added disruption of a second gene kills cells (Nijman, 2011). The first example of exploiting this approach in molecularly targeted cancer therapy was the inhibition of members of the enzyme family Poly (ADP-ribose) polymerase (PARP), key players involved in DDR, in *BRCA1/2* deficient tumors (Lord and Ashworth, 2017; Huang et al., 2020). In fact, this concept has been tested in several clinical trials (ClinicalTrials.gov Identifier: NCT00494234, NCT00494442) with great success. Because of the dynamic functions of HMTs and their heavy involvement in DNA damage, among other processes, they have been increasingly considered as druggable targets for discovery and pharmacological intervention of various cancers. Just in the past decade, pharmacological targeting of HMTs in the context of DDR has become a promising avenue for novel cancer therapies. It is worth noting, however, that although evidence suggests the exciting potential of novel therapeutic targets among HMTs, the field of epigenetic therapies has only recently started making significant progress toward improved targeting. Many issues remain to be resolved. For example, synergistic combinations of pharmacological HMT inhibitors and other treatment modalities, i.e., chemotherapy and radiation, still need to be evaluated and optimized for more beneficial clinical outcomes. Here, we briefly summarize several small molecule inhibitors targeting HMTs that have emerged in pre-clinical testing or early stages of clinical trials as part of promising standalone or combinatorial cancer treatments, including pharmacological inhibitors of DOT1L, G9a, EZH2, and PRMT5, and discuss their potential in combination strategies with canonical DNA damaging agents or DDR inhibitors (Figure 2 and Table 1).

**TABLE 1:** Small molecule inhibitors of DOT1L, G9a/GLP, EZH2, and PRMT5 and their anticancer activities in relation to DNA damage repair.

Target protein	Compound	Mode of action	Documented effects on DNA damage	Clinical trial
**DOT1L**	SGC0946	SAM-competitive	In colorectal cancer *in vitro*, treatment of SGC0946 resulted in decreased γH2AX levels, defective HR-mediated DSB repair (Kari et al., 2019)	N/A
	EPZ-5676 (Pinometostat)	SAM-competitive	In colorectal cancer *in vitro*, treatment of SGC0946 resulted in decreased γH2AX levels, defective HR-mediated DSB repair (Kari et al., 2019); Rectal cancer cells were sensitized to DNA damage-inducing chemotherapy and PARP inhibition following EPZ-5676 treatment (Amé et al., 2004; Liu et al., 2014)	Phase I trial completed in adult and pediatric patients with relapsed or refractory acute myeloid leukemia (AML) and acute lymphoblastic leukemia (ALL) harboring MLL gene rearrangements (ClinicalTrials.gov identifiers: NCT01684150, NCT02141828); Clinical trials currently ongoing to examine the combination of Pinometostat and standard DNA damage-inducing cancer treatment modalities (ClinicalTrials.gov identifiers: NCT03701295, NCT03724084)
	EPZ004777	SAM-competitive	N/A	N/A

**EZH2**	UNC1999	SAM-competitive	UNC1999 aggravated genotoxic effects induced by treatments of olaparib, an FDA approved PARP inhibitor in cells deficient in DDR pathways, enhancing its synthetic lethal effects in BRCA-deficient cell lines and AML patient cells (Caruso et al., 2018)	N/A
	EPZ-6438 (Tazemetostat)	SAM-competitive	Treatment of Tazemetostat sensitized PARPi effect in BRCA-defective cancer cells *in vitro* and *in vivo* (Yamaguchi et al., 2018)	Approved by FDA for patients 16 years and older with metastatic or locally advanced epithelioid sarcoma ineligible for complete resection as well as relapsed/refractory follicular lymphoma

**G9a/GLP**	UNC0638	Substrate-competitive	Treatment of U2OS cells with UNC0638 disrupted BRCA-BARD retention at DNA damage sites (Wu et al., 2015). The use of the PARP inhibitor Olaparib in combination with UNC0638 also resulted in a synergistic reduction of clonogenic survival in breast cancer cells (Carvalho et al., 2014)	N/A
	BIX-01294	Substrate-competitive	Loss of H3K9 methylation through G9a inhibition with BIX-01294 increased radiosensitivity of a panel of glioma cells (Gursoy-Yuzugullu et al., 2017)	N/A
	UNC0642	Substrate-competitive	G9a inhibition with UNC0642 conveyed a significant reduction in both NHEJ and HR repair in ovarian carcinoma cells (Watson et al., 2019)	N/A
	BRD4770	SAM-competitive	Combined inhibition of Checkpoint kinase 1 (Chk1), a key regulator of cell cycle transition in response to DNA damage, and G9a with BRD4770 disrupted pancreatic cancer cell growth, replication fork progression, and DNA damage signaling, ultimately leading to induction of cell death (Urrutia et al., 2020)	N/A

**PRMT5**	EPZ015666	Peptide-competitive	Loss of PRMT5 activity via EPZ015666 resulted in impaired HR, leading to DNA-damage accumulation, p53 activation, cell-cycle arrest, and cell death (Hamard et al., 2018)	N/A
	EPZ015938	Substrate-competitive	The combination of Gemcitabine and EPZ015938 resulted in synergistic accumulation of Gem-induced DNA damage in pancreatic cells *in vitro* and *in vivo* (Wei et al., 2020)	Phase I safety and clinical activity study underway in myelodysplastic syndrome and AML (ClinicalTrials.gov Identifier: NCT03614728); Phase I dose escalating study ongoing in solid tumors and non-Hodgkin lymphoma (ClinicalTrials.gov Identifier: NCT02783300)
	JNJ-64619178	SAM-competitive and peptide-competitive (simultaneous)	N/A	Phase I clinical trial continuing as a potential treatment for B cell non-Hodgkin lymphoma, lower risk MDS and advanced solid tumors (ClinicalTrials.gov Identifier: NCT03573310)

### DOT1L

A lot of small molecule inhibitors for DOT1L target the common cofactor-binding site for SAM within the methyltransferase structure (Yu et al., 2012). Utilizing structure-guided medicinal chemistry, EPZ004777, a SAM-competitive DOT1L inhibitor, was the first meaningful proof-of-concept for targeting any HMT (Daigle et al., 2011). Since then, SGC0946, a brominated analog of EPZ004777 that takes advantage of a hydrophobic cleft in DOT1L surrounding its adenine ring, and EPZ-5676, an aminonucleoside analog which became the first reported HMT inhibitor to enter human clinical trials, have been also developed (Daigle et al., 2013). Both molecules better occupy the SAM-binding pocket and disrupt its structural integrity via conformational rearrangement to improve inhibition of DOT1L over EPZ004777 (Yu et al., 2012).

As previously discussed, DOT1L is a key player in DSB repair via HR in several cancers. Depletion of DOT1L methyltransferase activity after SGC0946 and EPZ-5676 treatments leads to an impaired DNA damage response indicated by decreased γH2AX levels as well as defective HR-mediated DSB repair without affecting NHEJ in colorectal cancer cell lines (Kari et al., 2019). Continuous infusion of EPZ-5676 for 21 days in nude rat subcutaneous xenograft models of MLL-rearranged leukemia achieved well-tolerated, complete, and sustained tumor regression for more than 30 days post-treatment (Daigle et al., 2013). Reductions in both treatment duration and dose in the same model still sustained tumor regression, albeit with slightly lower efficacy.

EPZ-5676, also referred to as Pinometostat, has completed phase 1 trials in adult and pediatric patients with relapsed or refractory acute myeloid leukemia (AML) and acute lymphoblastic leukemia (ALL) harboring rearrangements of the MLL gene (ClinicalTrials.gov identifiers: NCT01684150, NCT02141828). These trials demonstrated only modest clinical efficacy (Stein et al., 2018). However, additional preclinical investigations have indicated that combination strategies of DOT1L inhibitors with chemotherapeutic agents or other chromatin modifying drugs may offer benefit (Klaus et al., 2014; Liu et al., 2014). Interestingly, inhibition of DOT1L sensitized MLL-rearranged leukemia cells to DNA damage-inducing chemotherapy by inhibiting their DNA damage response (Liu et al., 2014). In rectal cancer cells, the depletion of DOT1L has also recently been demonstrated to increase sensitivity to inhibition of PARP-1 (Kari et al., 2019). Pharmacological inhibition of PARP-1 delays DNA lesion repair and increases sensitivity to further damage. Taking this one step further, PARP inhibition may result in inadequate SSB repair (Dantzer et al., 2000). SSBs may then accumulate and result in DSBs, and if cells have any defects in DSB repair, they will face great, often fatal challenges to survive and proliferate. Cells with *BRCA1/2* mutations can be highly sensitive to further blockade of SSB repair via PARP inhibition due to their compromised ability to repair DSBs properly and efficiently via HR. These studies highlight the significant involvement of DOT1L in DDR and suggest great clinical potential for DOT1L inhibition in combination with DNA damaging chemotherapies. To date, the National Cancer Institute (NCI) has two clinical trials currently recruiting to examine the combination of Pinometostat and standard cancer treatment modalities (ClinicalTrials.gov identifiers: NCT03701295, NCT03724084). Thus, explorations for the optimal use of DOT1L inhibitors have just started.

### G9a

Various studies have identified G9a as a regulator of HR in response to DSB formation. In human cancers, the G9a complex is often recruited to chromatin and modulates efficient HR through its interaction with RPA. G9a deficiency has been shown to impair DDR and sensitize cancer cells to more DSBs by disrupting Rad51 and RPA foci formation in response to damage (Yang et al., 2017). BIX-01294, the first G9a complex inhibitor discovered through high through-put screening (Kubicek et al., 2007), is an H3 peptide substrate-mimetic molecule. While highly specific, this small molecule also shows cell toxicity not attributed to its inhibitory effects. Despite these shortcomings, however, BIX01294 offered hope to the treatment of various diseases mediated by this epigenetic pathway and provided a backbone for the design and synthesis of several subsequent G9a inhibitors, such as UNC0638, UNC0642, and more (Vedadi et al., 2011; Liu et al., 2013). In pre-clinical studies, loss of H3K9me through BIX-01294, UNC0638, and UNC0642 treatments hypersensitized tumor cells to DSB-inducing treatment modalities and resulted in inhibited DSB repair through, interestingly, both HR and NHEJ (Agarwal and Jackson, 2016; Gursoy-Yuzugullu et al., 2017; Watson et al., 2019). This may be related to the fact that most G9a inhibitors also target its complex partner, GLP, which has been implicated in NHEJ (Watson et al., 2019).

As mentioned, cells harboring *BRCA1/2* mutations are highly sensitive to defective DDR following PARP inhibition (Dantzer et al., 2000; Amé et al., 2004). BRCA1 must be recruited and retained at DNA damage sites for it to carry out its regulatory functions in HR (Scully et al., 1997). This process is dependent on the BRCT domains of BRCA1 (BRCA1-BRCT) and BRCA1 forming a complex with the BRCA1-associated RING domain (BARD1) protein (Wu et al., 1996). BARD1 has been shown to interact with H3K9me2 in response to DNA damage via direct binding of HP1, a H3K9 reader protein, to the BRCT domain of BARD-1 (Wu et al., 2015). Treatment of U2OS cells with H3K9 specific HMT inhibitor UNC0638 disrupted BRCA-BARD retention at DNA damage sites. In breast cancer, treatment with the PARP inhibitor olaparib in combination with UNC0638 also resulted in a synergistic reduction of clonogenic survival, suggesting that leveraging H3K9 methyltransferases as a target with PARP inhibitors in cancer might have therapeutic potential (Carvalho et al., 2014).

In terms of SAM-competitive small molecule G9a complex inhibitors, a notable example is BRD4770, which was discovered by Yuan et al. (2012). BRD4770 reduced cellular levels of H3K9me2 and H3K9me3, induced cell senescence, inhibited both anchorage-dependent and independent proliferation and resulted in G2/M cell cycle arrest in Panc-1, a pancreatic cancer cell line (Yuan et al., 2012). Work from our laboratory has shown that combined inhibition of Checkpoint kinase 1 (Chk1), a key regulator of cell cycle transition in response to DNA damage, with prexasertib, and G9a, using BRD4770, disrupted pancreatic cancer cell growth, replication fork progression, and DNA damage signaling, ultimately leading to induction of cell death (Urrutia et al., 2020), further supporting the strategy of DDR targeting in conjunction with G9a complex inhibition.

Although many potent G9a/GLP inhibitors have been discovered with promising results *in vitro* and progress has been made with some of these inhibitors for *in vivo* studies in recent years, no inhibitor has advanced to clinical trials so far due to challenges with pharmacodynamics and pharmacokinetics optimization (Vedadi et al., 2011; Liu et al., 2013). More studies are needed to augment pharmacodynamics and pharmacokinetics characteristics as well as toxicity profiles of current and future inhibitors. Due to the promise of this pathway as a target for anti-cancer agents, improved development and use of G9a inhibitors will continue to be in high demand.

### EZH2

EZH2 regulates the expression of many genes instrumental to lineage specification, cell cycle regulation, and DNA repair (Yamagishi and Uchimaru, 2017). Most of the EZH2 inhibitors confer their highly selective and potent inhibition through SAM competition via a conserved 2-pyridone core, the most notable being UNC1999 and EPZ-6438, also known as tazemetostat (Duan et al., 2020). So far, tazemetostat has been approved by FDA for patients 16 years and older with metastatic or locally advanced epithelioid sarcoma ineligible for complete resection as well as relapsed/refractory follicular lymphoma (U.S. Food and Drug Administration, and Center for Drug Evaluation and Research, 2020).

Interestingly, targeting EZH2 may promote synthetic lethality approaches in an HR-related capacity to improve the anti-tumor efficacy of PARP1 inhibition in *BRCA1/2*-deficient cancers. PARP1 interacts with and regulates EZH2 following alkylating DNA damage (Caruso et al., 2018). PARylation of EZH2, which is the addition of negatively charged ADP-ribose polymers in an enzymatic reaction, resulted in inhibition of EZH2 HMT activity (Masutani et al., 2003; Caruso et al., 2018). Caruso et al. (2018) also demonstrated that EZH2 inhibition via pharmacological inhibitor UNC1999 aggravated genotoxic effects induced by treatments of olaparib, an FDA approved PARP inhibitor, enhancing its synthetic lethal effects in BRCA-deficient cell lines and AML patient cells. As research ensues to illuminate the potential of targeting EZH2 as a part of combinatorial therapies with DDR inhibitors for more malignancies, more patients will be able to benefit from these new therapeutic strategies.

### PRMT5

Notably, PRMT5 acts as part of a multimeric complex with a variety of partner proteins that regulate its function and specificity (Antonysamy et al., 2012). One of its key interacting partners is MEP50, a WD-repeat–containing protein, which forms a (PRMT5)_4_(MEP50)_4_ octamer that has higher enzymatic activity than PRMT5 alone. Together, the PRMT5-MEP50 complex is regarded as the active “methylosome” *in vivo*, which is an important consideration for its therapeutic targeting. As described, PRMT5 is also a key player in DDR. It has been shown to cooperate with various factors to act as a wide-spectrum epigenetic regulator of DDR genes involved in HR, NHEJ, and G2 cell cycle arrest upon detection of DNA damage. In some studies, pharmacological targeting of PRMT5 decreased expression of some DDR genes and hindered DSB repair in multiple cancers *in vitro*, resulting in genomic instability, cell cycle defects, aberrant splicing of key DDR regulators, and ultimately DNA damage accumulation (Bedford, 2007; Hamard et al., 2018; Zhou et al., 2018; Checa-Rodríguez et al., 2020; Hwang et al., 2020).

The first pharmacological inhibitor of the PRMT5-MEP50 complex, EPZ015666, was discovered by Chan-Penebre et al. (2015). It binds to the peptide binding site of PRMT5 and has anti-proliferative effects on mantle cell lymphoma cell lines and xenograft models (Penebre et al., 2014; Chan-Penebre et al., 2015). GSK3326595 (EPZ015938) a substrate-competitive, improved PRMT5-MEP50 inhibitor, is currently under two clinical trials: a phase I safety and clinical activity study in myelodysplastic syndrome (MDS) and AML (ClinicalTrials.gov Identifier: NCT03614728); and a phase I dose escalating study in solid tumors and non-Hodgkin lymphoma (ClinicalTrials.gov Identifier: NCT02783300). JNJ-64619178, which inhibits the PRMT5 complex through simultaneously binding the SAM- and protein substrate-binding pockets, is also under phase I clinical trial as a potential treatment for B cell non-Hodgkin lymphoma, lower risk MDS and advanced solid tumors (ClinicalTrials.gov Identifier: NCT03573310). In 2019, PF-06939999 and PRT543, two of the latest PRMT5 complex inhibitors, also entered early phase clinical trials (ClinicalTrials.gov Identifier: NCT03854227, NCT03886831). Interestingly, the DOT1L inhibitor EPZ004777 also inhibits PRMT5, albeit at >1000-fold lower selectivity with an IC_50_ of ∼500nM against isolated PRMT5 (Daigle et al., 2011). However, this compound was later found inactive against PRMT5 in complex with MEP50 (Yu et al., 2012), suggesting that EPZ004777 is not effective to target the active PRMT5 complex *in vivo*.

Expression of *PRMT5* correlates with multiple gene players in the DDR pathway across various clinical cancer datasets and its depletion leads to accumulated DNA damage in cancers (Hamard et al., 2018; Hwang et al., 2020). Furthermore, targeting PRMT5 in the context of some of its non-histone substrates may also impact DDR integrity to offer additional therapeutic vulnerabilities. For instance, when DNA damage occurs, PRMT5 methylates p53 at residues R333, R335, and R337, which promotes p53 oligomerization and targeting to the nucleus (Jansson et al., 2008). Moreover, PRMT5 stimulates p53-mediated cell cycle arrest, while its depletion triggers p53-dependent apoptosis, suggesting that p53 methylation via PRMT5 plays a central role in determining the type of response to DNA damage. PRMT5 also methylates E2F-1, which is often phosphorylated by ATM/ATR and Chk1/Chk2 to augment apoptosis upon DNA damage (Cho et al., 2012). PRMT5-mediated methylation of E2F-1 negatively regulates its function and impacts protein stability. Depleting PRMT5 by siRNA resulted in stabilization of E2F-1 protein levels, an increase in E2F target gene expression, reduced growth rate and restored apoptosis. RAD9, a protein heavily involved in cell cycle checkpoint in response to DNA damage, was also reported to interact with and be methylated by PRMT5 (He et al., 2011). He et al. (2011) showed that PRMT5-mediated methylation of RAD9 at R172, R174, and R175 is required for cellular resistance to DNA damage, and loss of this methylation by alanine mutagenesis caused S/M and G2/M checkpoint defects in mouse embryonic stem cells. Thus, the concept of targeting PRMT5 in combination with DNA damage-inducing therapies, such as radiation or chemotherapy, or other DDR deficiencies is an exciting avenue for investigation.

In support of utilizing PRMT5 inhibitors within the context of DDR, pharmacological inhibition of PRMT5 sensitizes tumors to treatments inducing DDR that are otherwise prone to resistance (Hamard et al., 2018; Secker et al., 2019; Hwang et al., 2020). For example, treatment of osteosarcoma cells with PRMT5 inhibitors reduces 53BP1 protein levels upon DNA damage as well as enhances cell senescence mediated by chemotherapy such as cisplatin (Hwang et al., 2020; Li et al., 2020). An *in vivo* CRISPR screen using a pancreatic cancer orthotopic patient-derived xenograft model identified PRMT5 as a target to synergistically enhancing cytotoxicity of gemcitabine, a first- or second-line chemotherapy for pancreatic cancer (Wei et al., 2020). This is likely due to the accumulation of excessive DNA damage from impaired HR activities (Wei et al., 2020). Congruent with the demonstrated role for PRMT5 in HR and the sensitization of HR deficient cells to PARP inhibition, combined inhibition of PRMT5 and PARP has synergistic cytotoxic effects on AML cells while sparing normal hematopoietic cells (Hamard et al., 2018). These studies represent a promising new therapeutic approach to harness targeting DDR via an HMT such as PRMT5 for AML and likely offers the opportunity to serve as an effective approach in other cancers as well.

## Perspectives and Conclusion—Risk Assessment and Preparedness Plan

Histone methyltransferases have long been established as critical players in gene transcription, expression regulation, DNA damage repair, and many more processes instrumental to cell integrity and normal physiology. Many of the HMTs have also been validated and implicated as viable drug targets in emerging epigenetic cancer therapies. We reviewed prominent histone methylation marks involved in DDR pathways and the potential of their respective small molecule inhibitors as not just therapeutic targets, but also probes for further elucidating HMT functions in cancer epigenetic regulation. It is truly exciting that there has been a notable array of potent and selective inhibitors of HMTs, many of which have undergone rigorous pre-clinical studies and demonstrated clinical usefulness in the context of DNA damage.

Challenges remain, however, for targeting HMTs in DDR pathways effectively as cancer therapies. As histone methylation is fundamental in normal human physiology, inhibition of HMTs may lead to toxicities in patients. Therefore, toxicology studies are warranted to ensure safe clinical success. Finding potent small molecule inhibitors with low off-target effects is also a major challenge, as many HMTs share structural similarities as well as evolutionarily conserved domains and co-factors (Huang et al., 2020). Researchers in the field will need to complete rigorous examinations of pharmacokinetics, toxicities, functional validation, and medicinal chemistry profiles to confirm the roles of such inhibition in cancers and the validity of candidate compounds.

As drug resistance and heavy side effects from high drug doses become more persistent among cancer patients due to the overwhelming complexity of DDR pathways, combination therapy has become a promising approach to combat the issue, improve patients’ quality of life via more sparing treatment regimens, and increase efficacy of currently in-use treatment modalities. As reviewed here, we have seen tremendous progress in the discovery of novel, synergistic therapeutic combinations with inhibitors of HMTs and DDR pathway protein members.

While there remain gaps in knowledge regarding the complete “accident scene” as to whether some histone methylation events succumb to the fate of a collateral victim in the wake of DNA damage, there is evidence to support that certain HMTs play a more active role in the DDR process as part of the critical rescue team. Targeting HMTs in the context of DNA damage is a promising strategy for cancer therapeutics, although its promise lies in the ability for us to mechanistically study oncogenesis, as well as overcome drug resistance and high toxicity profiles by discovering and designing optimized, synergistic combination therapies. Ultimately, a multi-pronged approach to harness chromatin-related DDR effectors along with induction of DNA damage or inhibition of other key nodes in DDR pathways could offer a full arsenal of valuable strategies to destroy cancer cells.

## Author Contributions

LH searched the related published articles and wrote the original draft. GL designed and supervised the study, as well as revised the manuscript. Both authors contributed to the article and approved the submitted version.

## Conflict of Interest

The authors declare that the research was conducted in the absence of any commercial or financial relationships that could be construed as a potential conflict of interest.

## Publisher’s Note

All claims expressed in this article are solely those of the authors and do not necessarily represent those of their affiliated organizations, or those of the publisher, the editors and the reviewers. Any product that may be evaluated in this article, or claim that may be made by its manufacturer, is not guaranteed or endorsed by the publisher.
